# Опыт применения терипаратида для лечения послеоперационного гипопаратиреоза у пациентов, получающих заместительную почечную терапию программным гемодиализом

**DOI:** 10.14341/probl13075

**Published:** 2022-05-30

**Authors:** А. К. Еремкина, А. М. Горбачева, В. А. Ененко, Е. Е. Литвинова, Н. Г. Мокрышева

**Affiliations:** Национальный медицинский исследовательский центр эндокринологии; Национальный медицинский исследовательский центр эндокринологии; Клиника «Семейная»; Национальный медицинский исследовательский центр эндокринологии; Национальный медицинский исследовательский центр эндокринологии

**Keywords:** гемодиализ, гипопаратиреоз, терипаратид, остеопороз, клинический случай

## Abstract

Частота хронического послеоперационного гипопаратиреоза при выполнении тотальной паратиреоидэктомии по поводу вторичного и третичного гиперпаратиреоза у пациентов с терминальной почечной недостаточностью, по данным различных авторов, может достигать 20% и более. На фоне приема активных метаболитов витамина D, препаратов кальция не всегда удается достичь целевых значений кальция, поэтому возникает необходимость применения заместительной терапии рекомбинантным паратгормоном. Единственным препаратом данного ряда, одобренным и разрешенным Американским Агентством по контролю за пищевыми продуктами и медикаментами (FDA) и зарегистрированным в Российской Федерации, является терипаратид. Однако он зарегистрирован как анаболический антиостеопоретический препарат и не показан для лечения хронического гипопаратиреоза. Применение терипаратида при послеоперационном гипопаратиреозе у пациентов, получающих заместительную почечную терапию программным гемодиализом в Российской Федерации, ранее не изучалось, лимитированы данные по этому вопросу и в зарубежной литературе. Тем не менее это потенциальный вариант лечения гемодиализных пациентов с хроническим гипопаратиреозом и тяжелыми костными нарушениями. В настоящей статье будут рассмотрены 2 клинических случая, демонстрирующих возможности заместительной и анаболической терапии терипаратидом в данной когорте больных.

## Abstract (en)

The frequency of chronic postoperative hypoparathyroidism after total parathyroidectomy for secondary and tertiary hyperparathyroidism in patients with end-stage renal failure, according to various authors, can reach 20% or more. Prescribing active metabolites of vitamin D and calcium it is not always sufficient for achievement of target goals. This dictates the need for replacement therapy with recombinant parathyroid hormone. Teriparatide is the only drug of this series approved by the American Food and Drug Administration (FDA) and registered in the Russian Federation. However, it is registered as an anabolic anti-osteoporotic drug and is not indicated for the treatment of chronic hypoparathyroidism. The use of teriparatide in postoperative hypoparathyroidism in patients receiving renal replacement therapy with programmed hemodialysis in the Russian Federation has not been previously studied. Data on this issue is also limited in foreign literature. However, it is a potential treatment option for hemodialysis patients with chronic hypoparathyroidism and severe bone disorders. In this article, we present 2 clinical cases of substitution and anabolic therapy with teriparatide in this cohort of patients.

## АКТУАЛЬНОСТЬ

Минеральные и костные нарушения при хронической болезни почек (МКН-ХБП) — системное нарушение костно-минерального гомеостаза, обусловленное ХБП и проявляющееся одним из следующих признаков или их комбинацией: отклонениями впоказателях фосфорно-кальциевого обмена и развитием вторичного гиперпаратиреоза (ВГПТ), нарушениями обмена кости, ее минерализации, объема, линейного роста или прочности, внескелетной кальцификацией [1].

Распространенность ВГПТ у пациентов, получающих заместительную почечную терапию программным гемодиализом, в Европе и Австралии варьируется от 30 до 49%; в Северной и Южной Америке составляет порядка 54%, в Индии — 28%, вЯпонии — 11,5% [2]. По результатам международного проспективного когортного исследования Dialysis outcomes and practice patterns study (DOPPS) с 2012 по 2015 гг., включающего 20 612 пациентов в 543 учреждениях [3], самый низкий уровень интактного паратгормона (иПТГ) в сыворотке крови у пациентов на гемодиализе (600 пг/мл) был выявлен в Японии (1%), самый высокий — в Саудовской Аравии (27%) и России (30%), промежуточные показатели чаще регистрировались в странах Европы (8–21%).

Медикаментозная терапия ВГПТ направлена на достижение ключевых терапевтических целей — профилактику/коррекцию гиперфосфатемии, поддержание целевых показателей альбумин-скорректированного кальция, иПТГ, 25(ОН) витамина D, для чего используется большой пул лекарственных средств. Однако при длительной декомпенсации заболевания и гиперплазии околощитовидных желез (ОЩЖ) с узловой трансформацией и моноклональным ростом эти меры не способны замедлить прогрессирование ВГПТ [4][5], что приводит к резистентности к медикаментозной терапии и потребности в хирургическом лечении [6]. Для таких пациентов паратиреоидэктомия (ПТЭ) становится методом выбора [7][8], при этом оптимальным объемом оперативного вмешательства является субтотальная или тотальная ПТЭ с аутотрансплантацией ОЩЖ [9].

Учитывая большой объем ПТЭ, одним из самых частых его осложнений является послеоперационный гипопаратиреоз. По данным различных авторов [10–12], частота стойкого гипопаратиреоза после субтотальной паратиреоидэктомии колеблется в пределах от 2% до 17%; при выполнении тотальной ПТЭ частота гипопаратиреоза может превышать 20%. Риск развития данных осложнений коррелирует с квалификацией хирурга, а также с уровнем сложности выполняемой операции [13–15].

Для достижения нормокальциемии у пациентов с хроническим послеоперационным гипопаратиреозом, в том числе находящихся на гемодиализе, применяются активные метаболиты витамина D и препараты кальция. Современные рекомендации указывают на необходимость поддержания концентрации альбумин-скорректированного кальция для данной когорты больных в нижне-нормальном диапазоне или даже несколько ниже референсных значений при отсутствии клинических проявлений гипокальциемии. Данная рекомендация основана на целесообразности профилактики низкообменной формы почечной остеодистрофии, представляющей собой один из основных факторов риска переломов костей.

Несмотря на меры профилактики, не всегда удается добиться стабилизации параметров кальций-фосфорного обмена и адекватного костного обмена у пациентов с терминальной стадией ХБП и стойким гипопаратиреозом, что диктует необходимость применения заместительной терапии рекомбинантным ПТГ.

На сегодняшний день в клиническую практику внедрены два препарата этого класса: терипаратид (Forsteo®; Eli Lilly and Company, США; рекомбинантная 1–34 N-концевая последовательность человеческого ПТГ) и рекомбинантный паратгормон (Natpara®; рПТГ 1–84, Takeda, Япония). Также разработан препарат — аналог паратгормонподобных пептидов абалопаратид (Tymlos®; Radius Health, США). рПТГ 1–84 — единственный препарат, зарегистрированный для терапии хронического гипопаратиреоза [16–18]. Однако данное лекарственное средство, так и не прошедшее регистрацию в Российской Федерации, в сентябре 2019 г. было отозвано производителем с рынка из-за проблем со шприц-ручкой [19].

Терипаратид разрабатывался и зарегистрирован как анаболический антиостеопоротический препарат, и он не одобрен для лечения хронического гипопаратиреоза. Тем не менее имеется ряд клинических исследований, доказывающих его эффективность для лечения пациентов с различными формами хронического гипопаратиреоза без ХБП, проявляющимися в снижении потребности в препаратах кальция и витамина D, улучшении профиля кальциемии, кальциурии, микроархитектоники костной ткани и качества жизни [20].

Отдельно стоит упомянуть сложность диагностики и лечения костных нарушений при ХБП С3–5D. Так, ранее считалось, что оценка минеральной плотности костной ткани при помощи рентгеновской денситометрии не позволяет предсказывать остеопоротические переломы у пациентов с ХБП С3–5D, однако в редакции рекомендаций KDIGO 2017 г. денситометрия рассматривается уже как достаточно достоверный способ диагностики патологии костной ткани. Своеобразным золотым стандартом диагностики является биопсия костной ткани, однако ее рекомендовано применять в случае, если результаты смогут повлиять на принятие клинических решений (как, например, в случае диагностики адинамической костной болезни) [21].

Работы, посвященные применению терипаратида при послеоперационном гипопаратиреозе у пациентов, получающих заместительную почечную терапию программным гемодиализом, сильно лимитированы. Так, в Российской Федерации ранее не было описано ни одного клинического случая. Тем не менее это потенциальный вариант анаболического лечения пациентов со стойким послеоперационным гипопаратиреозом и тяжелыми костными нарушениями у пациентов с терминальной стадией ХБП. В настоящей статье будут рассмотрены 2 клинических случая, демонстрирующих возможности заместительной и анаболической терапии терипаратидом в данной когорте больных.

## ОПИСАНИЕ СЛУЧАЕВ

## Клинический случай 1.

Пациентка В., 56 лет, длительно наблюдалась по поводу двустороннего нефролитиаза, хронического пиелонефрита. В 2004 г. по экстренным показаниям начата заместительная почечная терапия программным гемодиализом. Резкое повышение уровня ПТГ до 1000 пг/мл было отмечено с 2009 г., проводилось лечение активными метаболитами витамина D, периодически цинакальцетом. 18.06.2014 ввиду стойкой декомпенсации ВГПТ (ПТГ до 1500 пг/мл) и значимой гиперплазии четырех ОЩЖ проведена тотальная ПТЭ. В послеоперационном периоде развился хронический гипопаратиреоз, назначалась терапия альфакальцидолом, препаратами кальция, фосфат-биндерами. При этом часто фиксировались гипокальциемические эпизоды ивыраженная гиперфосфатемия, которая ограничивала назначение препаратов витамина D.

Результаты физикального, лабораторного и инструментального исследования

В июне 2018 г. впервые была госпитализирована в отделение патологии околощитовидных желез ФГБУ «НМИЦ эндокринологии» Минздрава России. На момент поступления пациентка принимала альфакальцидол 0,5 мкг 3 раза в неделю, карбонат кальция (порошок) 2 г/сут, севеламер 2400–3200 мг/сут. Концентрация кальция в диализирующем растворе на момент поступления — 1,25 ммоль/л. При поступлении отмечались клинические признаки гипокальциемии. В анализах крови уровень альбумин- скорректированного кальция составил 2,07 ммоль/л, также отмечались гиперфосфатемия (2,23 ммоль/л), снижение ПТГ до 16 пг/мл. На фоне коррекции терапии с назначением 0,25 мкг/сут альфакальцидола, 2400 мг/сут севеламера, 3000 мг/сут карбоната кальция (таблетированных форм), 1200 МЕ/сут колекальциферола достигнуты целевые показатели (кальций, скорректированный на альбумин, 2,13 ммоль/л, фосфор 1,49 ммоль/л). Динамика показателей кальциево-фосфорного обмена и маркеров костной резорбции представлена в таблице 1.

**Table:** Таблица 1. Показатели сывороточных концентраций кальция, фосфора и маркеров костной резорбции пациентки В.Table 1. Serum concentrations of calcium and phosphorus and markers of bone resorption of patient B. during teriparatide therapy

	05.07.18	11.05.19	22.11.19	13.02.20	21.05.20	02.06.20
Кальций общий, ммоль/л (2,15–2,55)	2,07	2,11	2,11	2,31	1,64	2,13
Альбумин-скорректированный кальций, ммоль/л (2,15–2,55)	2,07	-	-	-	1,69	2,07
Фосфор, ммоль/л (0,74–1,52)	2,23	1,8	1,06	0,94	2,08	1,86
ЩФ, Ед/л (50–150)	228	-	-	-	252	117
Остеокальцин, нг/мл (11-43)	300	-	-	-	-	266,7
С-концевой телопептид коллагена I типа, нг/мл (0,01–0,69)	1,75	-	-	-	-	1,53

В ходе госпитализации пациентке впервые была проведена остеоденситометрия: диагностирован выраженный остеопороз поясничного отдела позвоночника и лучевой кости (табл. 2). По данным рентгенографии грудного и поясничного отделов позвоночника компрессионные переломы не определялись (рис. 1). Из дополнительных факторов риска развития остеопороза у пациентки имела место ранняя менопауза с 40 лет (заместительную гормональную терапию не получала).

**Table:** Таблица 2. Динамика минеральной плотности костей пациентки В.Table 2. Dynamics of bone mineral density of patient В.

Дата	МПК в поясничном отделе позвоночника, T-критерий (SD)
L1	L2	L3	L4	L1–L4
Июнь 2018 г.	-2,5	-2,7	-2,2	-3,2	-2,6
Июнь 2020 г.	-2,0	-2,0	-1,8	-2,6	-2,1
	МПК в бедренной кости, Т-критерий (SD)
Neck	Wards	Troch	Total
Июнь 2018 г.	-1,7	-1,8	-1,9	-1,6
Июнь 2020 г.	-1,4	-1,6	-1,8	-1,5
	МПК в дистальном отделе лучевой кости, T-критерий (SD)
Radius UD	Radius 33%	Radius total
Июнь 2018 г.	-4,2	-4,6	-5,1
Июнь 2020 г.	-4,7	-5,0	-5,4

**Figure:**
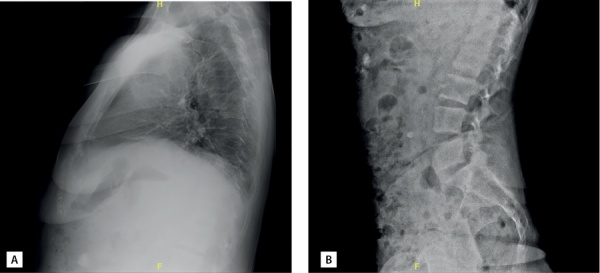
Рисунок 1. Рентгенография позвоночника пациентки В.: грудного (А) и поясничного (В) отделов в боковой проекции. Пояснения в тексте.Figure 1. X-ray of patient В’s spine: thoracic (A) and lumbar (B) sections in lateral view. Explanations in the text.

Лечение

С учетом высокого риска переломов и наличия хронического гипопаратиреоза была инициирована терапия терипаратидом в дозе 20 мкг/сут.

Дополнительным фактором, обуславливавшим необходимость назначения терипаратида, стало наличие внескелетной кальцификации. Так, при эхокардиографии выявлялось уплотнение с включениями кальция стенок корня и восходящего отдела аорты, кольца и створок аортального клапана, створок митрального клапана.

К сожалению, в связи с проблемами в получении терипаратида инъекции препарата выполнялись с некоторыми пропусками — с ноября 2018 г. по март 2019 г., и затем в течение года с марта 2019 г. по апрель 2020 г. Параллельно продолжался прием альфакальцидола 0,25 мкг/сут, карбоната кальция в дозах 1500–3000 мг/сут (3000 мг — вне терапии терипаратидом), севеламера 2400 мг/сут. На фоне инъекций терипаратида удавалось достичь стойкой нормокальциемии, нормофосфатемии (см. табл. 1). Субъективно пациентка отмечала ­улучшение общего состояния, уменьшение болей в костях, спине, расширение двигательной активности. Переломов за время терапии не возникло. На фоне отмены терипаратида в мае 2020 г. вновь развились гипокальциемия и гиперфосфатемия.

Исход и результаты последующего наблюдения

Повторная госпитализация в отделение в июне 2020 г. К ведущим жалобам при поступлении относились затруднение при самостоятельном передвижении в связи с болезненностью в правой пяточной области, выраженная общая слабость. Намомент поступления концентрация кальция в диализирующем растворе составляла 1,5 ммоль/л. По результатам рентгеновской денситометрии (через 16 мес от начала терапии терипаратидом) была отмечена значительная положительная динамика минеральной плотности кости (МПК) в поясничном отделе позвоночника и проксимальном отделе бедренной кости, умеренная отрицательная динамика в левой лучевой кости (см. табл. 2). Компрессионные переломы исключены по данным рентгенографии.

Из анамнеза стало известно, что в апреле 2020 г. возникли жалобы на боли в покое в мышцах правой голени и стопе, раневой дефект правой стопы. По данным проведенного обследования (ангиография, транскутанная оксиметрия) была диагностирована окклюзия поверхностных бедренных артерий с развитием критической ишемии обеих конечностей вследствие атеросклероза, кальцификации артерий нижних конечностей. 29.04.2020 выполнена баллонная ангиопластика подколенной, передней большеберцовой, поверхностной бедренной и малоберцовой артерий справа, впоследствии пациентка получала двойную антитромботическую терапию. Несмотря на проведенное лечение, в июне 2020 г. снова появились признаки перемежающейся хромоты и раневой дефект правой стопы. При осмотре определялся сухой некроз пяточной области Wagner II справа. По данным дуплексного сканирования артерий нижних конечностей были выявлены стеноз обеих поверхностных бедренных артерий (до 80%), окклюзия артерий голени с обеих сторон, ввиду отсутствия критической ишемии нижних конечностей, а также низкой ожидаемой эффективности повторной баллонной ангиопластики было рекомендовано наблюдение в динамике.

Учитывая положительную динамику основных показателей минерального обмена, прогрессирующую внескелетную кальцификацию, пациентке было рекомендовано продолжить терапию терипаратидом в дозе 20 мкг п/к ежедневно в комбинации сальфакальцидолом (0,25 мкг/сут), карбонатом кальция (1000–1500 мг/сут), севеламером (2400 мг/сут) и колекальциферолом 1000 МЕ/сут.

## Клинический случай 2.

Пациентка А., 41 год, впервые поступила в отделение патологии околощитовидных желез в сентябре 2017 г. с жалобами на боли в суставах, мышцах, периодически возникающие судороги в кистях и стопах, снижение роста на 10 см в течение 1,5–2 лет. При осмотре обращала на себя внимание выраженная кифосколиотическая деформация позвоночника. Из анамнеза известно, что в возрасте 2 лет у пациентки были выявлены пузырно-мочеточниковый рефлюкс и хронический рецидивирующий пиелонефрит; в 1987 г. перенесла операцию по поводу стриктуры уретры; в 1989 г. оперирована по поводу пузырно-мочеточникового рефлюкса. 19.09.2001 г. планово переведена на заместительную почечную терапию программным гемодиализом.

Повышение сывороточной концентрации ПТГ отмечала с 2011 г. (максимально до 1817 пг/мл), гиперплазия ОЩЖ диагностирована в 2016 г. Проводилась терапия цинакальцетом, препаратами активного и нативного витамина D без достижения целевых показателей фосфорно-кальциевого обмена. Пациентка перенесла множественные низкоэнергетические переломы — обеих ключиц, правой лучевой кости, ребер, шейки левой бедренной кости. Также в анамнезе был асептический некроз шеек обеих бедренных костей, по поводу которого проводилось двустороннее эндопротезирование тазобедренных суставов. Эндопротезирование левого сустава осложнилось перипротезным переломом и потребовало повторного хирургического вмешательства.

В связи с резистентностью к консервативной терапии, тяжелыми костными нарушениями в октябре 2017 г. в ­выполнена тотальная ПТЭ с аутотрансплантацией ткани ОЩЖ в мышцу левого предплечья. В послеоперационном периоде у пациентки развилась острая гипокальциемия (кальций общий 1,56 ммоль/л, кальций ионизированный 0,78 ммоль/л), потребовавшая проведения инфузионной терапии с глюконатом кальция. В последующем переведена на терапию по схеме альфакальцидол 2 мкг/сут, колекальциферол 2000 МЕ/сут, ацетат кальция 1300–1500 мг/сут, гидроксикарбонат магния 705 мг/сут с умеренной положительной динамикой (табл. 3). Концентрация кальция в диализирующем растворе на момент госпитализации в 2018 г. составляла 1,75 ммоль/л.

**Table:** Таблица 3. Динамика сывороточных показателей минерального обмена пациентки А.Table 3. Serum concentrations of mineral metabolism markers of patient A.

	18.09.2017	Хирургическое лечение 03.10.2017	05.10.2017	29.06.2018	04.07.2018	Начало терапии терипаратидом — апрель 2019 г.	12.05.2019	01.09.2019	27.09.2019	02.10.2019	03.09.2020	07.09.2020	10.09.2020
Кальций общий, ммоль/л (2,15–2,55)	2,05	1,56	1,65	1,76	1,91	1,89	1,93	2,46	2,23	3,09	2,83
Кальций ионизированный, ммоль/л (1,03–1,29)	1,02	0,78									
Альбумин-скорректированный кальций, ммоль/л (2,15–2,55)	2.13	1,62	1,65	1,78			1,89	2,46	2,19	3,03	2,73
Фосфор, ммоль/л (0,74–1,52)	1,54		0,75	0,9	0,79	0,89	0,95	0,95	2,03	1,03	1,17
ПТГ, пг/мл (15–65)	3395	8,05	48,23						15,49		
ЩФ, Ед/л (50–150)	2071		180				229		78		
Остеокальцин, нг/мл (11–43)			168				125,5		232,3		
С-концевой телопептид коллагена I типа, нг/мл (0,01–0,69)			1,22				2,05		2,19		

Результаты физикального, лабораторного и инструментального исследования

При госпитализации в 2018 г. установлен диагноз хронического гипопаратиреоза в стадии декомпенсации (альбумин-скорректированный кальций 1,65 ммоль), снижение сывороточной концентрации ПТГ сохранялось и после перевода пациентки нагемодиализ с концентрацией кальция в диализирующем растворе 1,25 ммоль/л. При рентгенографии грудного и поясничного отделов позвоночника в боковой проекции в июле 2018 г. выявлена деформация по типу «рыбьих» позвонков, компрессионные переломы тел ThX (35% потери МПК), ThIX (40%), ThVIII (65%), ThVII (68%) позвонков и потеря МПК в задних третях тел поясничных позвонков максимально до 37% (рис. 2 А, В).

**Figure:**
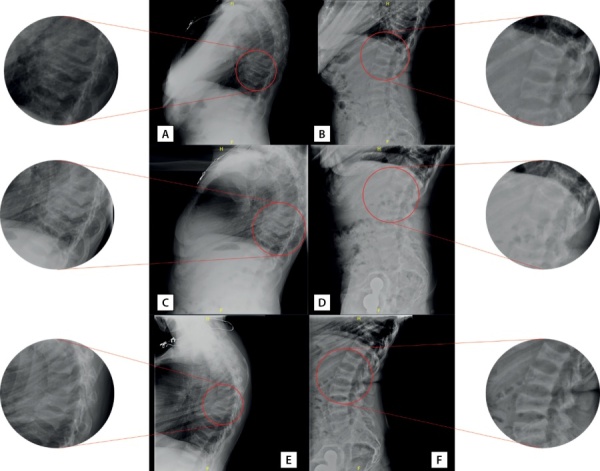
Рисунок 2. Рентгенография позвоночника пациентки А.: грудной (А, C, E) и поясничный (В, D, F) отделы в боковых проекциях. А и В — июль 2018 г., C и D — сентябрь 2019 г., Е и F — сентябрь 2020 г. Пояснения в тексте.Figure 2. X-ray of patient A’s spine: thoracic (A, C, E) and lumbar (B, D, F) sections in lateral views. A and B — July 2018, C and D — September 2019, E and F — September 2020. Explanations in the text.

Лечение

Принимая во внимание высокий риск развития переломов, пациентке с хроническим гипопаратиреозом и выраженной гипокальциемией в качестве безальтернативного метода лечения был рекомендован терипаратид. Продолжен гемодиализ с концентрацией кальция в диализирующем растворе 1,25 ммоль/л. С апреля 2019 г. пациентка проводила регулярные инъекции препарата в дозе 20 мкг/сут в сочетании с альфакальцидолом 1 мкг/сут, карбонатом кальция 3 г/сут, колекальциферолом 2000 МЕ/сут. На этом фоне отмечалось значимое улучшение общего самочувствия (уменьшение слабости, мышечных судорог), лабораторных показателей (табл. 3), прироста МПК (табл. 4).

**Table:** Таблица 4. Динамика минеральной плотности костей пациентки А. Cентябрь 2017 г. — до паратиреоидэктомии (Hologic); июнь 2018 г. — 8 мес после паратиреоидэктомии, до начала терапии терипаратидом (Hologic); октябрь 2019 г. — суммарно 6 мес терапии терипаратидом (Hologic); сентябрь 2020 г. — суммарно 18 мес терапии терипаратидом (Lunar iDXA).Table 4. Dynamics of bone mineral density of patient A. September 2017 — before parathyroidectomy (Hologic); June 2018 — 8 months after parathyroidectomy, before starting teriparatide (Hologic); October 2019 — a total of 6 months of teriparatide therapy (Hologic); September 2020 — a total of 18 months of teriparatide therapy (Lunar iDXA).

Дата	МПК в поясничном отделе позвоночника, Z-критерий (SD)
L1	L2	L3	L4	L1–L4
Сентябрь 2017 г.	-1,9	-2,7	-3,9	-4,2	-3,3
Июнь 2018 г.	0,1	-0,9	-2,4	-2,2	-1,5
Октябрь 2019 г.	0,6	0,4	-0,1	0,2	0,2
Сентябрь 2020 г.	3,6	3,4	3,9	3,9	3,8
	МПК в дистальном отделе лучевой кости, Z-критерий (SD)
Radius UD	Radius 33%	Radius total
Сентябрь 2017 г.	-0,9	-3,8	-2,9
Июнь 2018 г.	-0,4	-3,3	-2,1
Октябрь 2019 г.	0,8	-1,9	-0,7
Сентябрь 2020 г.	3,2	-1,9	-0,3

Осенью 2019 г. при рентгенографии грудного и поясничного отделов позвоночника в боковой проекции определялись компрессионные переломы c ThVI до ThX (15–29% потери массы, преимущественно вентрально/каудально), LIII и LV (25–30% потери массы в дорсальных третях позвонков) (рис. 2 С, D). По результатам эхокардиографии 01.10.2019 определялись признаки кальцификации кольца и створок аортального и митрального клапанов. Ранее эхокардиография пациентке не выполнялась.

Учитывая хороший эффект по результатам DEXA, сохранение тенденции к гипокальциемии (низкоцелевой уровень кальция), было принято решение продолжить терапию терипаратидом.

Исход и результаты последующего наблюдения

Контрольное обследование проведено в сентябре 2020 г. Кроме терипаратида, на момент поступления пациентка получала карбонат кальция 3000 мг/сут, альфакальцидол 1 мкг/сут, колекальциферол 2000 МЕ/сут. На этом фоне была зафиксирована гиперкальциемия, что потребовало снижения дозы альфакальцидола до 0,5 мкг/сут в междиализные дни, карбоната кальция — до 2000 мг/сут. Для коррекции гиперфосфатемии инициирована терапия севеламером в дозе 2400 мг/сут.

По результатам рентгенденситометрии сохранялась положительная динамика прироста МПК в лучевой кости (см. табл. 4). Обращал на себя аномальный прирост МПК в поясничном отделе позвоночника. При контрольной рентгенографии отмечено снижение высоты тел ThXII (16% потери массы), ThXI (14%), ThX (23%), ThIX (20%), ThVIII (49%), ThVII (45%), ThVI (43%), ThV (44%), LV (32%), LIV (26%), LIII (22%), LII (18%), LI (15%) позвонков (рис. 2 Е, F). Однако на рентгенограммах отмечалось появление симптома «сэндвича» (или «джемпера регбиста») — значимое утолщение кортикального слоя в телах позвонков. С одной стороны, была отмечена отрицательная динамика в виде появления дополнительного снижения высоты тел ранее поврежденных и прежде интактных позвонков. С другой стороны, учитывая выраженную кифотическую деформацию позвоночника пациентки, указанные изменения могли быть следствием как усиления степени кифоза, так и боковой ротации отдельных позвонков. По решению врачебной комиссии в связи с отсутствием альтернативных методов лечения остеопороза у данной пациентки рекомендовано продолжить инъекции терипаратида в течение последующих 6 мес (до достижения суммарного срока в 2 года).

## ОБСУЖДЕНИЕ

Лечение минеральных и костных нарушений у пациентов с хроническим гипопаратиреозом и терминальной стадией почечной недостаточности представляет сложную проблему и требует персонализированного подхода.

Терипаратид не зарегистрирован для лечения хронического гипопаратиреоза, однако вопрос о его назначении активно встает при сочетании недостаточной функции ОЩЖ и тяжелого остеопороза [22]. Анаболический эффект терипаратида на кости особенно актуален для пациентов на гемодиализе с послеоперационным гипопаратиреозом, для которых крайне распространены костные нарушения по типу низкообменнной почечной остеодистрофии. По данным F Barreto и cоавт., остеопороз регистрируется у 46% диализных пациентов [23], при этом применение антирезорбтивных препаратов зачастую невозможно из-за выраженной гипокальциемии и низкого костного метаболизма.

У описанных нами пациенток на фоне ежедневных инъекций терипаратида удалось достичь целевых значений альбумин-скорректированного кальция и фосфора. Кроме того, с учетом этиопатогенетического действия терипаратида [24][25], данная терапия ожидаемо сопровождалась снижением доз пероральных препаратов кальция и витамина D, что особенно важно для пациентов с внескелетной кальцификацией

В течение первых месяцев терапии терипаратидом происходит стимуляция остеоида костными многоклеточными единицами, при этом образование кости значительно превышает ее резорбцию [26][27]. После инициации терапии рекомбинантным ПТГ у пациентки В. определялась положительная динамика МПК в осевых отделах скелета (до уровня остеопении), переломы отсутствовали. У пациентки А. через 18 мес терапии показатели МПК характеризовались аномально высокими значениями, однако это могло быть обусловлено не только развитием новых компрессионных переломов, но и ротацией отдельных позвонков в сочетании с увеличением минеральной плотности кортикального слоя. Представленные клинические случаи демонстрируют важность комплексной оценки состояния костной ткани, включая проведение традиционной рентгенографии. При возникновении новых переломов при рентгенденситометрии могут определяться «ложно завышенные» показатели, не отражающие истинную динамику костного обмена. Кроме того, при подозрении на возникновение новых компрессионных переломов у пациентов с выраженной кифосколиотической деформацией необходимо проводить рентгенографию не только в боковой, но и в прямой проекции для оценки степени ротации отдельных позвонков. Необходимо отметить, что у пациентки А. фиксировалось увеличение толщины кортикального слоя, что, вероятно, можно расценить как следствие применения терипаратида. В ряде исследований был продемонстрирован анаболический эффект терипаратида на кортикальный компонент крупных трубчатых костей (бедренная, плечевая), губчатой подвздошной кости. Однако количество работ, посвященных влиянию терипаратида на кортикальный компонент позвонков, ограничено, хотя это тоже может иметь важное значение в поддержании механической прочности позвоночника в целом [28].

Chen и соавт. продемонстрировали, что ежедневное введение терипаратида в течение 18 мес обезьянам cynomolgus, подвергшимся овариэктомии, сопровождалось увеличением толщины кортикальной оболочки поясничных позвонков. И это увеличение было связано с повышением их прочности при сжатии [29].

Безусловно, для оценки состояния костной ткани у пациентов с МКН-ХБП золотым стандартом остается проведение биопсии, однако данный метод диагностики был недоступен для наших пациентов. Вероятно, эффективность терипаратида в предотвращении новых переломов в данной группе пациентов сильно зависит от сроков назначения препарата (до развития множественных низкоэнергетических переломов). Однако этот вопрос остается открытым.

Результаты первых 6 мес лечения у наших пациентов в целом сопоставимы с данными открытого проспективного исследования Cejka D. и соавт. [30]. Через полгода лечения терипаратидом в дозе 20 мкг в сочетании с препаратами кальция и активными метаболитами витамина у 7 гемодиализных пациентов был достигнут значительный прирост МПК в поясничном отделе позвоночника (0,885±0,08 против 0,914±0,09 г/см2, p

Примечательно, что в литературе встречается только 1 публикация, Sumida К. и соавт. [31], в которой проводилась оценка изменений в лучевой кости у гемодиализных пациентов, получавших терипаратид. Всего было включено 22 пациента с хроническим послеоперационным гипопаратиреозом и низкой костной массой, инъекции терипаратида выполнялись в дозе 56,5 мкг 1 раз в неделю в течение 2 лет. Согласно полученным результатам, МПК увеличилась только в поясничном отделе позвоночника (на 3,3±1,9% через 24 нед терапии, на 3,0±1,8% через 48 нед терапии). В шейке бедра и дистальной трети лучевой кости определялась тенденция к снижению МПК, однако эти изменения не достигли статистической значимости.

Умеренная отрицательная динамика в лучевой кости у пациентки В. может быть обусловлена несколькими причинами. В исследовании женщин с тяжелым постменопаузальным остеопорозом без ХБП и ежедневным приемом терипаратида в дозе 20 мкг [32] значительная потеря МПК в дистальной трети лучевой кости по сравнению с исходным уровнем определялась на 18 и 24 мес терапии. Это объясняется особенностями самой ткани: ремоделирование в кортикальной кости происходит с образованием «корковой пористости» — своеобразных пустот, которые могут занижать значения МПК при денситометрии [27]. Кроме того, для ПТГ-опосредованного костеобразования необходима механическая нагрузка на кость, активирующая клетки-предшественницы надкостницы [33–35]. Это подтверждается исследованием Cohen A. и соавт. [36]. В данном исследовании значительное увеличение МПК наблюдалось в дистальной части большеберцовой кости, но отсутствовало в дистальном отделе лучевой кости. Таким образом, необходимо продолжить наблюдение для оценки отсроченного влияния терипаратида на лучевую кость.

У гемодиализных пациентов практически в 90% случаев [37][38] возникают специфические изменения сердечно-сосудистой системы вследствие эктопической кальцификации и кальцифилаксии как в исходе ВГПТ, так и на фоне массивной терапии препаратами кальция и активных метаболитов витамина D [39–42]. В представленных нами случаях у обеих пациенток диагностирован кальциноз сердечных клапанов без образования гемодинамически значимых стенозов. Кроме того, пациентка В. перенесла баллонную ангиопластику артерий нижних конечностей ввиду мультифокального атеросклероза и выраженной кальцификации сосудов. После реваскуляризации полного разрешения симптомов не отмечалось, сохранялась окклюзия задней большеберцовой артерии. В данном случае применение терипаратида может иметь дополнительные преимущества в замедлении процессов кальцификации как за счет уменьшения нагрузки препаратами кальция и метаболитами витамина D, так и за счет прямых эффектов препарата. В исследовании на мышах с сахарным диабетом и дефицитом рецепторов липопротеинов низкой плотности было показано, что ежедневные инъекции терипаратида приводят к снижению степени кальцификации как аортального, так и сердечного клапана. Также было обнаружено, что терипаратид увеличивает экспрессию остеопонтина — мощного циркулирующего ингибитора кальцификации [43].

В нашем исследовании пациенты не отмечали побочных реакций, что учитывалось в принятии решения о продолжении терапии рекомбинантным ПТГ. По данным литературы, наиболее часто при использовании терипаратида отмечались ортостатическая гипотензия, головокружение и судороги в ногах; тошнота и головная боль, транзиторная гиперкальциемия, носящая дозозависимый характер [31][44][45]. В настоящее время имеется ограничение в допустимой длительности применения препарата до 24 мес, что связано с противоречивыми результатами исследований на животных [45, 46]. В работе Vahle J.L. и соавт. были зафиксированы случаи остеосаркомы у крыс при применении препарата более 2 лет. При этом следует отметить, что за более чем 10-летний период назначения терипаратида в США и Европе ни одного случая остеосаркомы у человека зарегистрировано не было. Использование терипаратида у детей с генетически детерминированным гипопаратиреозом в течение 10 лет также не сопровождалось развитием остеосаркомы [47].

У обеих пациенток на фоне комбинированной терапии улучшилось общее самочувствие, расширилась физическая активность. Это подтверждает выводы исследователей о том, что терипаратид снижает инвалидизацию и улучшает качество жизни упациентов, получающих заместительную терапию программным гемодиализом [30][48].

## ЗАКЛЮЧЕНИЕ

Терипаратид может быть рассмотрен в качестве патогенетической заместительной и анаболической антиостеопоротической терапии у пациентов с МКН-ХБП, находящихся на лечении программным гемодиализом и имеющих хронический послеоперационный гипопаратиреоз. Использование препарата возможно по жизненным показаниям в случае отсутствия альтернативных методов лечения. На фоне терапии требуется комплексная оценка не только параметров кальций-фосфорного обмена, но и состояния костной ткани, включая традиционную рентгенографию. Необходимы дальнейшие рандомизированные исследования для оценки безопасности и эффективности назначения терипаратида в отношении данной когорты больных.

## ДОПОЛНИТЕЛЬНАЯ ИНФОРМАЦИЯ

Источники финансирования. Работа проведена в рамках выполнения Государственного задания Минздрава России (АААА-А20-120011790168-2).

Конфликт интересов. Авторы декларируют отсутствие явных и потенциальных конфликтов интересов, связанных с содержанием настоящей статьи.

Участие авторов. Еремкина А.К. — концепция и дизайн исследования, получение и интерпретация результатов; написание рукописи; Горбачева А.М. — концепция и дизайн исследования, получение и интерпретация результатов; написание рукописи; Ененко В.А. — получение и интерпретация результатов; написание рукописи; Литвинова Е.Е. — получение и интерпретация результатов; внесение в рукопись существенных правок; Мокрышева Н.Г. — концепция и дизайн исследования, получение и интерпретация результатов; внесение в рукопись существенных правок. Все авторы одобрили финальную версию статьи перед публикацией, выразили согласие нести ответственность за все аспекты работы, подразумевающую надлежащее изучение и решение вопросов, связанных с точностью или добросовестностью любой части работы.

Согласие пациента. Пациенты подписали добровольное информированное согласие на публикацию персональной медицинской информации в обезличенной форме в журнале «Проблемы эндокринологии».
